# Phototrophic Fe(II) oxidation benefits from light/dark cycles

**DOI:** 10.1111/1758-2229.13239

**Published:** 2024-03-15

**Authors:** Verena Nikeleit, Linda Roth, Markus Maisch, Andreas Kappler, Casey Bryce

**Affiliations:** ^1^ Department of Geosciences University of Tübingen Tübingen Germany; ^2^ Cluster of Excellence: EXC 2124: Controlling Microbes to Fight Infections Tübingen Germany; ^3^ School of Earth Sciences University of Bristol Bristol UK

## Abstract

Phototrophic Fe(II)‐oxidizers use Fe(II) as electron donor for CO_2_ fixation thus linking Fe(II) oxidation, ATP formation, and growth directly to the availability of sunlight. We compared the effect of short (10 h light/14 h dark) and long (2–3 days light/2–3 days dark) light/dark cycles to constant light conditions for the phototrophic Fe(II)‐oxidizer *Chlorobium ferrooxidans* KoFox. Fe(II) oxidation was completed first in the setup with constant light (9 mM Fe(II) oxidised within 8.9 days) compared to the light/dark cycles but both short and long light/dark cycles showed faster maximum Fe(II) oxidation rates. In the short and long cycle, Fe(II) oxidation rates reached 3.5 ± 1.0 and 2.6 ± 0.3 mM/d, respectively, compared to 2.1 ± 0.3 mM/d in the constant light setup. Maximum Fe(II) oxidation was significantly faster in the short cycle compared to the constant light setup. Cell growth reached roughly equivalent cell numbers across all three light conditions (from 0.2–2.0 × 10^6^ cells/mL to 1.1–1.4 × 10^8^ cells/mL) and took place in both the light and dark phases of incubation. SEM images showed different mineral structures independent of the light setup and ^57^Fe Mössbauer spectroscopy confirmed the formation of poorly crystalline Fe(III) oxyhydroxides (such as ferrihydrite) in all three setups. Our results suggest that periods of darkness have a significant impact on phototrophic Fe(II)‐oxidizers and significantly influence rates of Fe(II) oxidation.

## Graphical abstract

Light/dark periods happen daily in nature but are seldom included in experimental designs of phototrophic Fe(II)‐oxidizers like *Chlorobium ferrooxidans* KoFox. We investigated how different light/dark cycles impact Fe(II) oxidation rates, cell growth and Fe(III) mineral formation of these bacteria. We found that imitating a day/night cycle increased the maximum Fe(II) oxidation rates significantly and suggest that this could lead to an under estimation of anoxygenic Fe(II)‐oxidizers in the environment.

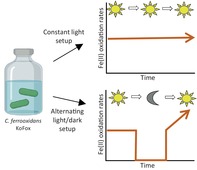

## INTRODUCTION

Iron is ubiquitous in the environment and influences most other major nutrient cycles: acting as a source or sink for heavy metals, carbon, nutrients and/or toxins during mineral formation and dissolution (Eickhoff et al., 2014; Kappler et al., 2021; Mu et al., 2016; Tipping, 1981). One kind of bacteria that can facilitate Fe(II) oxidation are anoxygenic phototrophic Fe(II)‐oxidizers (photoferrotrophs) like *Chlorobium ferrooxidans* KoFox (Heising et al., 1999; Widdel et al., 1993). These anoxygenic phototrophs produce ATP via the light reaction with visible light and generate NADPH to be used in the dark reaction to fix CO_2_ and build biomass. The latter can occur during both light and dark periods. To generate NADPH, different electron donors like organic carbon (acetate and glucose) and other inorganic substrates like H_2_, H_2_S^−^ or thiosulfate can be used by photoferrotrophs as alternatives to Fe(II) (Croal et al., 2009; Melton et al., 2014). For Fe(II) oxidation, photoferrotrophs can use dissolved Fe(II), i.e. Fe^2+^_aq_, or some poorly crystalline Fe(II) minerals like siderite, FeS and Fe(II)/Fe(III) mixed minerals like green rust and magnetite (Byrne et al., 2015; Han et al., 2020; Kappler & Newman, 2004).

The process of Fe(III) mineral formation by photoferrotrophic bacteria is complex and a combination of thermodynamic, kinetic, and biochemical factors control the final mineral products (Bryce et al., 2018; Kappler et al., 2021). Another important parameter that affects the activity of photoferrotrophs is light. Previous studies have investigated the importance of different light intensities and wavelengths for photoferrotrophic growth and mineral formation (Hegler et al., 2008; Schmidt et al., 2021). However, in all of these studies so far, light exposure duration was always kept constant during the incubation. Cultures were either exposed continuously to light or kept in the dark serving as a control. To represent environmentally relevant conditions, the impact of diurnal or changing illumination conditions needs to be considered in laboratory studies. Therefore, the present study aims to understand how different light/dark cycles that simulate the natural day/night cycle influence Fe(II) oxidation, Fe(III) mineral formation, and growth of photoferrotrophs.

## EXPERIMENTAL PROCEDURES

### Experimental design

*C. ferrooxidans* KoFox was used as a photoferrotrophic model strain and grown anoxically in serum vials with a 90:10 N_2_/CO_2_ headspace according to Heising et al. (1999) and Widdel et al. (1993). Three different light/dark setups were tested: constant light (CL, permanent illumination), long light/dark cycle (LC, alternating every 2–3 days), and short light/dark cycle (SC, 10 h light and 14 h dark). Serum bottles were placed at random in an incubator and incubated at 20°C. A 46 W (2700 K) light bulb with a wavelength range of 400–1000 nm was used as a light source, with an intensity of 20 to 23 μmol/sm^2^ photons. All setups were performed in biotic triplicates with one abiotic control. Slurry samples were taken in an anoxic glovebox for quantification of Fe and cells. Samples for mineral analyses were collected at the end of the experiment.

### Ferrozine assay

Fe(II) and Fe(total) were quantified spectrophotometrically with the ferrozine assay after Hegler et al., 2008. During sampling, 0.1 mL of slurry sample was added to 0.9 mL 1 M HCl, and the samples were stored at 4°C until quantification. The ferrozine‐Fe(II) complex was quantified at 562 nm using a spectrometer (Thermo Scientific Multiscan, Thermo Fisher Scientific). Measurements were conducted in triplicates.

### Cell quantification via flow cytometry

For flow cytometry, 180 μL of samples were fixed with 20 μL formaldehyde solution (CH_2_O). Minerals were dissolved by adding 200 μL of sterile, anoxic 100 mM Fe(II) ethylenediammonium sulphate tetrahydrate (Fe‐EDAS, FeSO_4_ *C_2_H_8_N_2_H_2_SO_4_*4H_2_O) and 600 μL oxalate solution (0.23 M (NH_4_)_2_C_2_O_4_*H_2_O and 0.17 M C_2_H_2_O_4_, pH 7). After 10 minutes, samples were transferred to a 96‐well plate for cell quantification using a flow cytometer equipped with an emission filter 585/15 nm (Attune Nxt flow cytometer, Thermo Fisher Scientific). Phototrophic cells were distinguished from noise or debris by gating based on their properties in the side scatter and YL 4 channel due to the autofluorescence from the cells (with emission filter 585/15 nm). Additionally, cells were also stained with a BacLight Green stain (Thermo Fisher Scientific, 1 μL stain/1 mL sample) and measured with BL laser (with emission filter 530/30 nm). The cell numbers obtained were the same as when using autofluorescence (Figure S1). The total number of events that show yellow fluorescence in the side scatter region associated with cells was divided by the total volume of sample run to give a final cell concentration in cells per millilitre. All measurements were conducted in triplicates and the results were reported as an average.

### Mössbauer spectroscopy

Slurry samples were passed through a filter (ø13 mm; 0.45 μm, nitrocellulose, Merck) which was then sealed between two pieces of airtight Kapton tape and kept frozen anoxically at −20°C until measurement. The sample was transferred to the instrument and only removed from the freezer before loading the samples inside the closed‐cycle exchange gas cryostat (Janis cryogenics). Absorption spectra were collected at 77 K and 5 K with a constant acceleration drive system (WissEL) in transmission mode with a ^57^Co/Rh source which was calibrated against a 7 μm thick α‐^57^Fe foil measured at room temperature. All spectra were analysed using Recoil (University of Ottawa) by applying a Voight Based Fitting site analysis. The half‐width at half maximum was fixed to a value of 0.124 mm/s for all sample analyses.

### Scanning electron microscope

Minerals were the main focus of this measurement and therefore cells were destroyed in the process. First, the slurry samples were washed to remove salts from the media. The sample was centrifuged at 20,000 rcf for 5 min, then the supernatant was removed, filled with MilliQ again, and repeated three times. MilliQ was then added to suspend the minerals. Roughly 100 μL washed sample was transferred onto a carbon‐coated sticker and left to dry at room temperature. Samples were placed on aluminium stubs and sputter‐coated at a working distance of 35 mm at 20 mA for 70 s to receive a 13 nm coating (BAL‐TEC SCD 005). Microscopy was conducted with a ZEISS FIB Crossbeam 550 L using an electron high tension of 2 kV, a working distance of 1.5 mm, and a secondary electron secondary ion (SESI) detector.

### Statistical analysis

Welsh *t*‐test was performed to calculate significant differences with a threshold of *α* = 0.05 for unequal variances between each sample.

## RESULTS AND DISCUSSION

### Effect of light/dark cycles on photoferrotrophic growth

**FIGURE 1:**
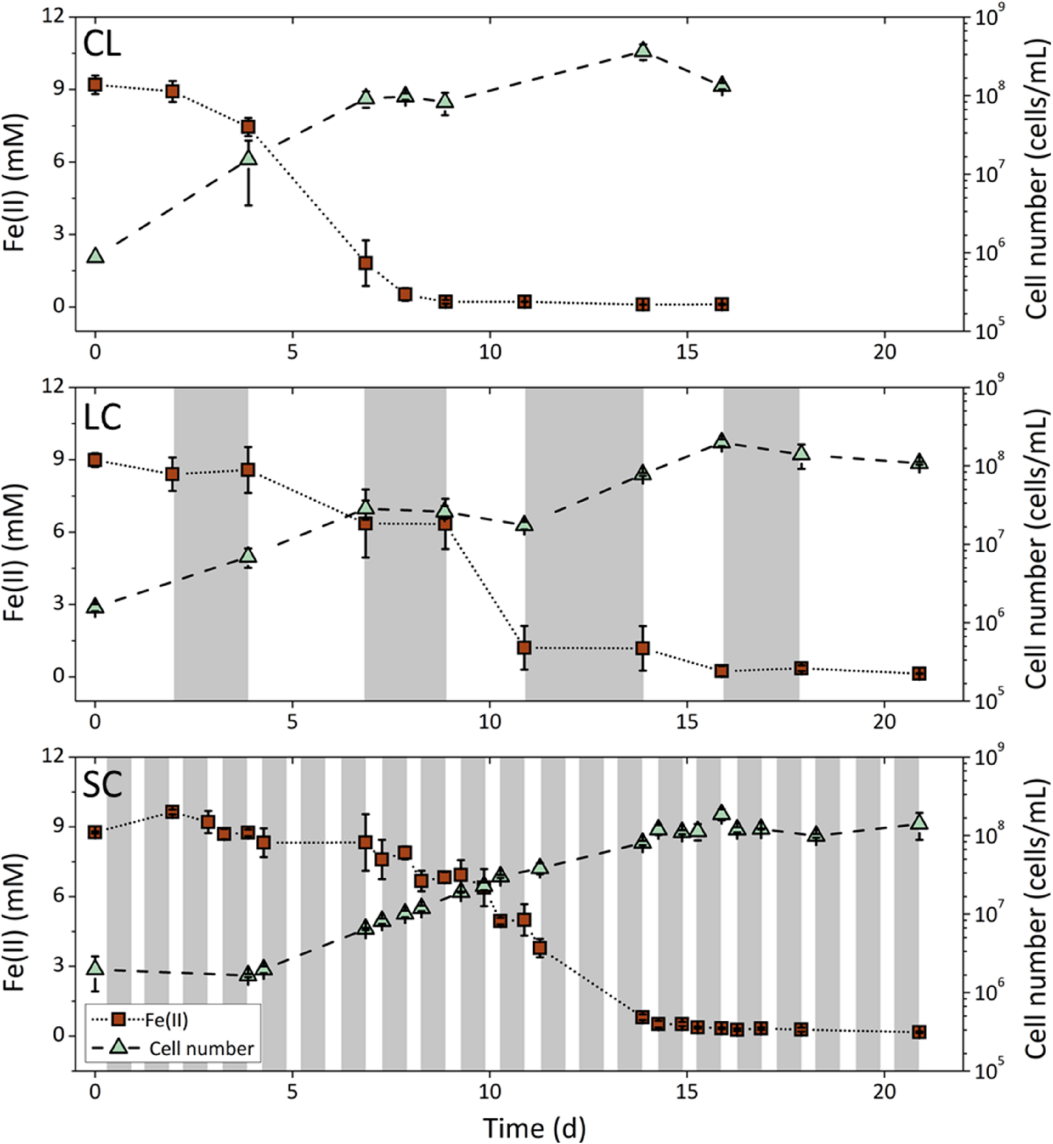
Figures show Fe(II) and cell numbers (averages of triplicates) of *Chlorobium ferrooxidans* KoFox grown in three different light conditions: Constant light (CL), long light/dark cycle (LC, alternating every 2–3 days) and short light/dark cycle (SC, 10 h light and 14 h dark). Dark periods are coloured grey.

The influence of light/dark cycles on Fe(II) oxidation was assessed by monitoring total Fe(II) and cell numbers. All Fe(II) (9 mM) was completely (98%) oxidised and cell numbers reached a plateau at the end of the incubations in all setups. Differences in the duration of Fe(II) oxidation and Fe(II) oxidation rates were observed in the different setups. Fe(II) oxidation started immediately in the constant light (CL) setup and was completed after 8.9 days. Cell numbers increased from 8.7 ± 0.2 × 10^5^ cells/mL to 8.2 ± 2.8 × 10^7^ cells/mL at the end of Fe(II) oxidation, and increased further even after all Fe(II) was oxidised to 1.3 ± 0.1 × 10^8^ cells/mL at 15.9 days (Figure 1). In the long/dark (LC) cycle where light/dark conditions were switched every 2–3 days, no lag phase in Fe(II) oxidation was observed and Fe(II) was almost completely oxidised (98%) after 15.9 days. During dark cycles, no Fe(II) oxidation was observed, i.e. during the periods of 2.0–3.9 days, 6.9–8.9 days, and 10.9–13.9 days. For cell numbers, no clear effect between light and dark conditions were observed and a steady cell number increase was observed until cells reached a constant number after 20.9 days (from 1.6 × 10^6^ ± 0.1 × 10^3^ cells/mL to 1.1 ± 0.04 × 10^8^ cells/mL).

In the short light/dark cycle setup (10 h light/14 h dark), the timing of Fe(II) oxidation varied in the triplicates to some extent. Different Fe(II) oxidation lag phases were observed ranging from 3.9 to 7.3 days while Fe(II) oxidation in these setups was completed by 20.9 days (98%). The lag phase was also observed in cell numbers, which started to increase after 3.9 days along with the start of Fe(II) oxidation and increased steadily until reaching a constant number at the end of the experiment after 20.9 days (1.4 ± 0.5 × 10^8^ cells/mL). In these setups, Fe(II) oxidation also halted during the dark phases, i.e. during the periods of 3.3–3.9 days, 8.3–8.9 days, 10.3–10.9 days, and 14.3–14.9 days (Figure 1).

It also appears that in the beginning the duration of light exposure is responsible for the lag phase in the short light/dark cycle. In the case of the continuous and long light/dark setup, no lag phase in Fe(II) oxidation and cell growth could be observed. Whereas, we could see a lag phase in the short light/dark setup from 3.9 to 7.3 days. The lag phase could be attributed to the short duration of the light period for the phototrophic bacteria to produce enough ATP and oxidise Fe(II) for cell growth. This could suggest that at the beginning of cell growth, it would be beneficial for the cultures to be exposed for longer in the light.

### Effect of light/dark cycles on Fe(II) oxidation rates

**FIGURE 2:**
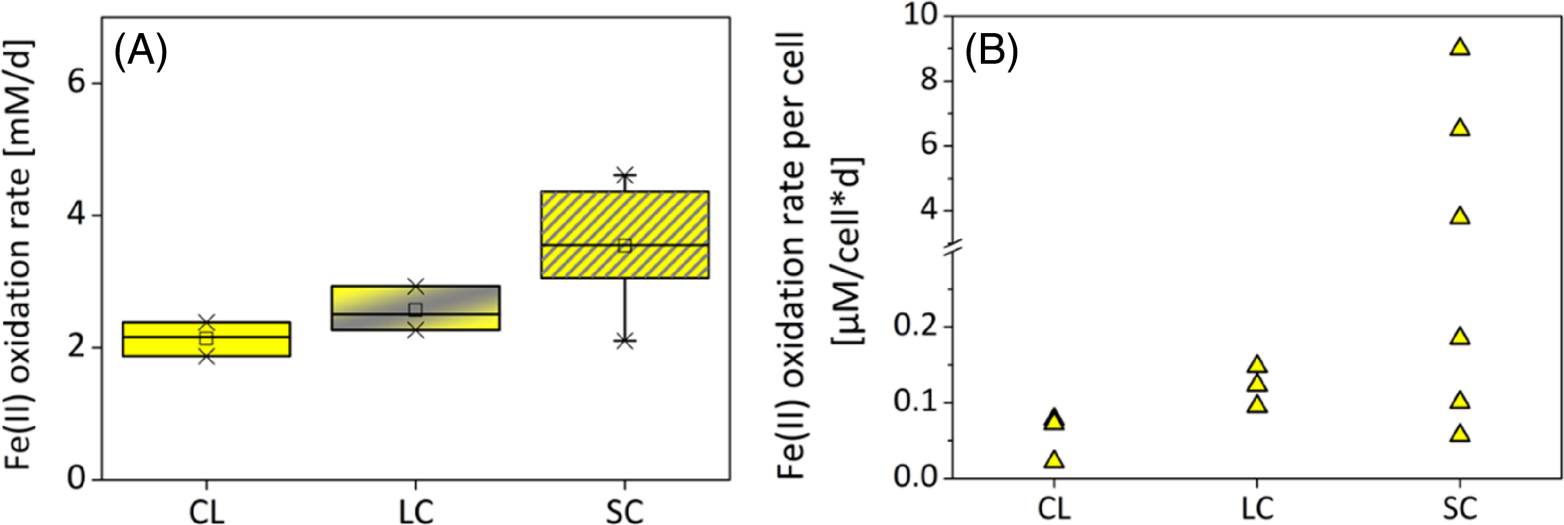
Panel A shows the maximum Fe(II) oxidation rates of *Chlorobium ferroooxidans* KoFox for constant light (CL), long light/dark cycle (LC), and short light/dark cycle (SC) setups. Rates were calculated from triplicates for CL and LC, for SC six rates were chosen. Welsh *t*‐test showed significant differences between the CL and the SC (*t* − 3.35, df 6.21, *p*‐value 0.015). Panel B shows the maximum Fe(II) oxidation rate per cell. The time points chosen were the same as in Panel A.

For the setup with constant light, maximum Fe(II) oxidation rates were calculated between the two fastest time points for each microcosm individually and averaged among all replicates (3.9–6.9 days). Maximum Fe(II) oxidation rates for the long light/dark cycle were calculated from the light phases that showed the fastest Fe(II) oxidation rate for each bottle and averaged (8.9–10.9 days). In the last setup (short light/dark cycle) the maximum rates from two light phases for each bottle were selected thus, in this case, mean rates are derived from six values (values for triplicate A and B were calculated from rates between 9.9–10.3 days and 10.9–11.3 days whereas triplicate C's rates were calculated from the period 3.9 to 4.3 days and 10.9 to 11.3 days). An alternative calculation method for the rates resulted in similar results and can be found in Figure S2. Here, Fe(II) concentrations from 9.8 d to 13.3 d were taken and divided by the time the sample was in the light. No Fe(II) concentration changes were observed in the abiotic bottles under any condition (not shown). The lowest maximum Fe(II) oxidation rates occurred in the constant light setups with a rate of 2.1 ± 0.3 mM/d. For the long light/dark setup, the maximum Fe(II) oxidation rate in the light was 2.6 ± 0.3 mM/d and the highest rate in the short light/dark setup was 3.5 ± 1.0 mM/d (Figure 2). A clear trend to higher Fe(II) oxidation rates with light/dark cycles was observed and in the case of the short light/dark cycles Fe(II) oxidation rates were significantly higher than in the constant light setup (*t* − 3.35, df 6.21, *p*‐value 0.015). The Fe(II) oxidation rates of *C. ferrooxidans* KoFox in the constant light setup were in the range of values from previous studies conducted under similar conditions (0.6–2.4 mM/d; continuously illuminated; Gauger et al., 2016; Hegler et al., 2008).

We also calculated the maximum Fe(II) oxidation rates per cell by using the Fe(II) oxidation rates and cell numbers. Cell numbers were calculated for the appropriate time interval for each Fe(II) oxidation rate. For Fe(II) oxidation rates per cell, similar trends were observed. The continuously illuminated setup showed the lowest Fe(II) oxidation rates (0.06 ± 0.03 μM/(cell*d)), followed by the long light/dark setup (0.12 ± 0.03 μM/(cell*d)) and the highest rates in the short light/dark setup (3.27 ± 3.83 μM/(cell*d)). In the setups with the short light/dark cycles, the rates per cell varied considerably. The lowest Fe(II) oxidation rates per cell measured in these setups were comparable to the continuous and long light/dark cycle but at three periods the Fe(II) oxidation rates/cell reached values up to 8.99 μM/(cell*d), which is 150 times higher than in the continuous light setup.

### Advantages of a light/dark lifestyle

These results indicate that a light/dark lifestyle is beneficial for phototrophic Fe(II) oxidation in terms of increasing the maximum Fe(II) oxidation rates. A trait that remained despite years of continuous cultivation under light conditions in the laboratory. In previous studies, the effect of light/dark cycles on non‐Fe(II)‐oxidising anoxygenic phototrophs was investigated and beneficial effects on biomass, protein, and coenzyme Q10 production were found (Hauruseu & Koblízek, 2012; Zhi et al., 2019). For *C. ferrooxidans* KoFox, no increase in biomass production (cell growth) but an increase in maximum Fe(II) oxidation rates were found with light/dark cycles.

The fact that the final cell numbers were similar in all can be linked to the experimental setup in the closed system. In each setup, the same amount of Fe(II) was present and oxidised (~98% of 9 mM). Given that the electrons from Fe(II) oxidation are directly coupled to CO_2_ fixation this has to lead to the same amount of biomass, independent of oxidation rates (of course variations in amounts of EPS, cellular proteins, carbohydrates, lipids, or pigments are possible). Interestingly, cell growth not only took place during the light periods but continued in the dark, suggesting that the cells used the ATP and NADPH produced in the light during dark periods to fix CO_2_.

One disadvantage of continuous light conditions could be that there is too much light which could lead to photodamage. If too many photons are absorbed and not used in the dark reaction, which is slower than the light reaction, reactive oxygen species can be created and damage bacteriochlorophylls and proteins (Wydrzynski & Hillier, 2011). One way to reduce this is by quenching chlorosomes, FMO (Fenna–Matthews–Olson) antenna protein, quenching of carotenoids with chlorophyll and with carotenoids which can also scavenge singlet oxygen directly (Blankenship et al., 1993; Blankenship & Matsuura, 2003; Foote, 1968; Renger & Wolff, 1977; Tsukatani et al., 2010; Wen et al., 2010). This could have occurred here with *C. ferrooxidans* KoFox and thus more energy would have been needed to produce the additional proteins/pigments and slowed down the maximum Fe(II) oxidation rates. *C. ferrooxidans* KoFox may use dark phases to utilise excessive photons, repair photodamage, and produce pigments needed for photosynthesis.

### Mineral identity and surface morphology

Identification of the minerals by ^57^Fe Mössbauer spectroscopy showed no difference between the minerals formed during Fe(II) oxidation in the different light illumination setups (Figure S3 and Table S1). The dominant iron mineral phase in all cases was a short‐range ordered Fe(III) mineral, such as the Fe(III) oxyhydroxide ferrihydrite (~50%), and an alternative Fe(III) phase (potentially poorly crystalline lepidocrocite) identified at 77 K (approx. 50%). Measurements at 5 K suggested very low particle size and/or low crystallinity. The formation of poorly crystalline Fe(III)‐phases such as ferrihydrite has been shown in previous studies with *C. ferrooxidans* KoFox (Kappler & Newman, 2004).

**FIGURE 3:**
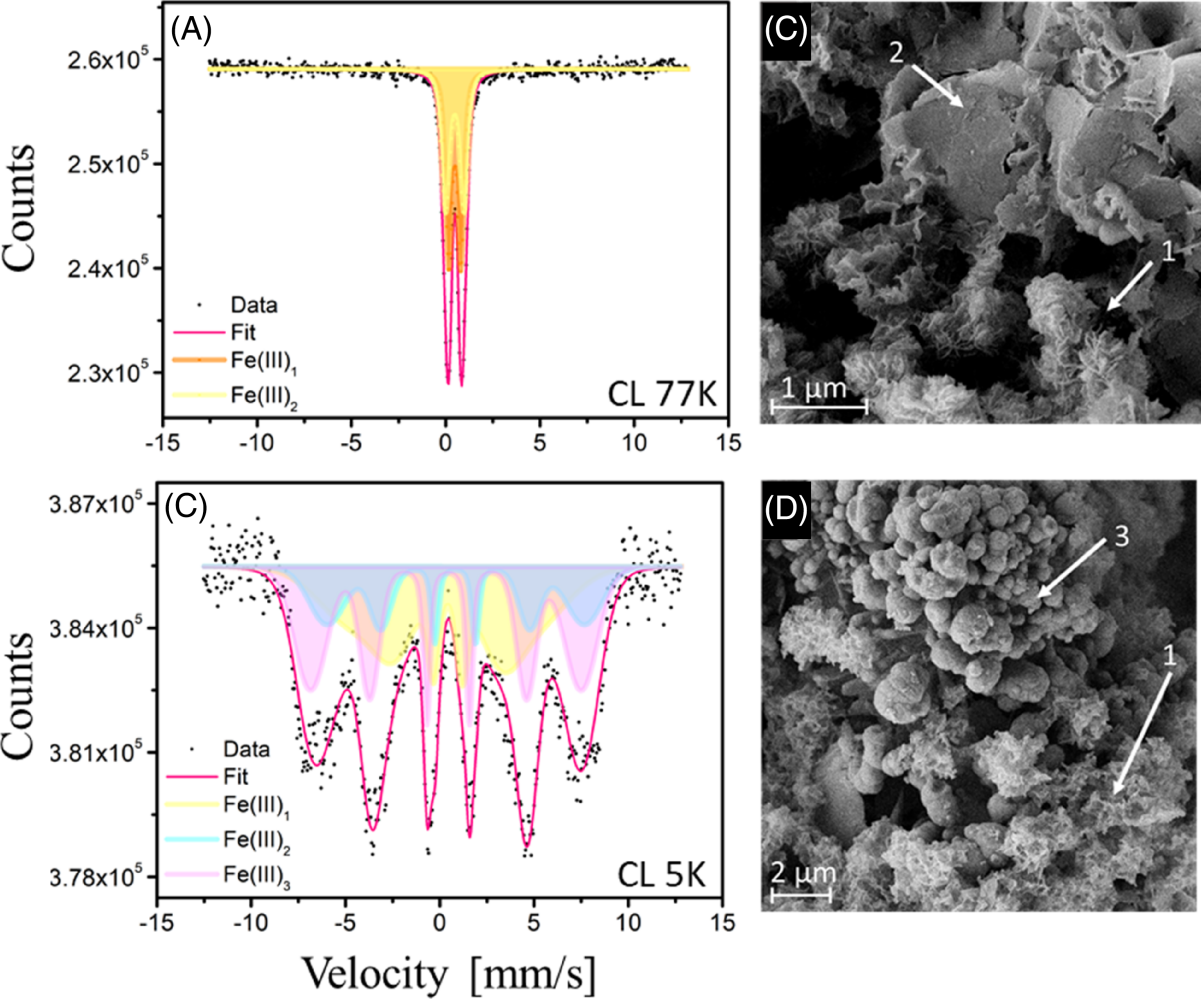
Mössbauer spectroscopy of C. ferroooxidans KoFox taken at 77 K and 5 K for constant light (A, C). SEM images of *Chlorobium ferrooxidans* KoFox grown under different light/dark conditions. Representative images show different mineral surfaces that were present in all setups representative of long light/dark cycle (C, D). Different mineral morphologies were detected: (1) fine flakes; (2) flaky; (3) rhombic.

We also analysed the morphology of the mineral aggregates using SEM and found that the mineral aggregates looked similar in the different light illumination setups. Three different morphologies, resembling fine flakes, flaky and rhombic structures dominated the three setups (Figures 3). This suggests that whilst light/dark cycles influence the physiology of photoferrotrophs, they have no significant influence on the identity and morphology of Fe(III) minerals.

## CONCLUSIONS

Our results show that the metabolic activity of phototrophic Fe(II)‐oxidizers varies with different diurnal cycles. This results in higher maximum Fe(II) oxidation rates during short light/dark cycles (10 h light/14 h dark). The influence of diurnal cycles is rarely investigated in the lab but should be considered when phototrophic Fe(II) oxidation rates are derived from experiments performed under continuous illumination and further extrapolated to quantify their impact on iron cycling in the environment. Overlooking the impact of diurnal cycling may result in the contribution of anoxygenic phototrophic Fe(II) oxidation being underestimated. We suggest further that cultivating phototrophic Fe(II)‐oxidizers (and phototrophs in general) in light/dark cycles is essential to better understand their physiology and environmental behaviour.

## AUTHOR CONTRIBUTIONS

**Verena Nikeleit:** Conceptualization (equal); data curation (lead); formal analysis (lead); investigation (lead); methodology (lead); validation (equal); visualization (equal); writing – original draft (lead); writing – review and editing (lead). **Linda Roth:** Formal analysis (supporting); investigation (supporting); methodology (supporting); visualization (supporting); writing – review and editing (supporting). **Markus Maisch:** Formal analysis (supporting); investigation (supporting); methodology (supporting); resources (supporting); writing – review and editing (supporting). **Andreas Kappler:** Conceptualization (equal); funding acquisition (supporting); methodology (supporting); project administration (supporting); resources (supporting); supervision (supporting); writing – review and editing (supporting). **Casey Bryce:** Conceptualization (equal); data curation (supporting); formal analysis (supporting); funding acquisition (lead); investigation (supporting); methodology (supporting); project administration (lead); resources (lead); supervision (lead); validation (supporting); visualization (supporting); writing – original draft (supporting); writing – review and editing (supporting).

## CONFLICT OF INTEREST STATEMENT

The authors declare no conflict of interest.

## Supporting information


**Figure S1.** Flow cytometry plots. Panels A and B show counts and laser (BL1‐blue laser; YL4‐yellow laser), and Panel C combines blue and yellow laser. In panels D and E side scatter is plotted with one laser (BL or YL).
Figure S2. Panel A shows the maximum Fe(II) oxidation rates of C. ferroooxidans KoFox for constant light (CL), long cycle (LC), and short cycle (SC) setups. Rates were calculated from triplicates for CL and LC, for SC 6 rates were chosen. SC+ was calculated from 9.8–13.8 days and divided by the light days.
Figure S3. Mössbauer spectroscopy of C. ferroooxidans KoFox taken at 77 K and 5 K for constant light (CL), short light/dark cycle (SC), and long light/dark cycle (LC).
Table S1. Summary of the Mössbauer parameters obtained by fitting. CS, centre shift; QS, quadrupole splitting; ε, quadrupole shift; H, hyperfine field; sigma, standard deviation of H; Pop, site population, i.e. relative spectral area. Db, doublet; S (Coll), collapsed sextet; S (Wide), widened sextet; S (Def), defined narrow sextet; χ^2^, goodness of the fit; Fe phase, identified iron phase. Samples were analysed for constant light (CL), short light/dark cycle (SC), and long light/dark cycle (LC).

## Data Availability

The data used to prepare the plots in figures 1 and 2 are available in Supplementary Data File 1.
